# Laparoscopic distal gastrectomy for gastric cancer patient with intestinal malrotation: report of a case

**DOI:** 10.1186/s40792-019-0606-9

**Published:** 2019-03-25

**Authors:** Susumu Inamoto, Kazutaka Obama, Satsuki Asai, Rei Mizuno, Yoshiro Itatani, Kyoichi Hashimoto, Shigeo Hisamori, Shigeru Tsunoda, Koya Hida, Kenji Kawada, Yoshiharu Sakai

**Affiliations:** Department of Surgery, Graduate School of Medicine, Kyoto University, 54 Shogoin- Kawara-cho, Sakyo-ku, Kyoto, 606-8507 Japan

**Keywords:** Gastric cancer, Congenital anomalies, Intestinal malrotation, Laparoscopic distal gastrectomy, Roux-en-Y reconstruction

## Abstract

**Background:**

Intestinal malrotation, which arises from incomplete rotation of the embryonic midgut, is one of the congenital anomalies usually diagnosed in infancy. On the other hand, intestinal malrotation detected in asymptomatic adults is very rare. It is frequently diagnosed incidentally during abdominal surgery. We report a case of asymptomatic intestinal malrotation diagnosed during laparoscopic distal gastrectomy for gastric cancer.

**Case presentation:**

A 59-year-old female was diagnosed with early-stage gastric cancer during health screening and admitted to our hospital for radical surgical treatment. Physical examinations and blood tests revealed nothing of note. The type 0-IIc gastric cancer was located in the posterior wall of the mid-body of the stomach. The histological type was poorly differentiated adenocarcinoma.

Esophagogastroduodenoscopy and computed tomography (CT) suggested that the depth of tumor invasion was the submucosal layer without regional lymph node swelling. The clinical stage according to the TNM 7th edition was cT1b N0 M0, cStage I.

Laparoscopic distal gastrectomy with D1+ lymph node dissection and Billroth-I method reconstruction was planned. During the infrapyloric lymph node dissection, a part of the pancreatic head showed unusual adherence to the first part of the duodenal wall. For safe and accurate lymphadenectomy while avoiding pancreatic injury, we deliberately focused on tracing the dissectible layer between the pancreatic parenchyma and fatty tissues including lymph nodes.

Also, we changed the reconstruction procedure from Billroth-I to Roux-en-Y. After distal gastrostomy, we could not find the ligament of Treitz or jejunum on the left side below the transverse colon. Based on a review of the CT image, this patient was diagnosed with intestinal malrotation.

Although the detection of malrotation during the operation was incidental, we could complete radical surgery and Roux-en-Y reconstruction safely. The type of malrotation was non-rotation (90°). She was discharged from our hospital without any complications.

**Conclusion:**

We encountered a case of adult asymptomatic intestinal malrotation with gastric cancer. Even when encountering such a case during laparoscopic gastrectomy, reviewing CT images carefully to reconsider the anatomical anomalies, and tracing the dissectible layer accurately with adequate countertraction can facilitate safe and successful surgery.

## Background

Gastric cancer is the fifth most common cancer and the third leading cause of cancer death worldwide. Annually, it is diagnosed in approximately 950,000 people globally and causes an estimated 723,000 deaths [1]. Even though chemotherapy or immunotherapy against gastric cancer has been gradually advanced, surgical treatment is still the most effective option.

Conventional radical treatment for early gastric cancer, which could not be treated by endoscopic mucosal resection (EMR) or endoscopic submucosal dissection (ESD), was previously surgical gastrectomy via open laparotomy. However, in the last 20 years, minimally invasive surgery for gastric cancer, such as laparoscopic gastrectomy, has rapidly developed [2, 3]. The advantages of laparoscopic surgery are as follows: it provides a magnified view, it allows sharing of the surgical field among surgeons, only a small incision is necessary, and the associated postoperative recovery is faster. On the other hand, it takes a longer operative time, increases medical costs, and requires special skills to handle laparoscopic devices and forceps.

Intestinal malrotation, which arises from incomplete rotation of the embryonic midgut, is one of the congenital anomalies usually diagnosed in infancy. Asymptomatic intestinal malrotation occurs in 1 in 500 live births [4], while symptomatic malrotation occurs in 1 in 6000 to 10,000. The most common symptoms are volvulus, intestinal obstruction, internal hernia, and superior mesenteric artery syndrome. On the other hand, intestinal malrotation detected in asymptomatic adults is very rare. It is frequently diagnosed incidentally during abdominal surgery. We report a rare case of asymptomatic intestinal malrotation diagnosed during laparoscopic distal gastrectomy for gastric cancer.

## Case presentation

A 59-year-old female was diagnosed with early-stage gastric cancer during health screening and admitted to our hospital for radical surgical treatment.

**Fig. 1:**
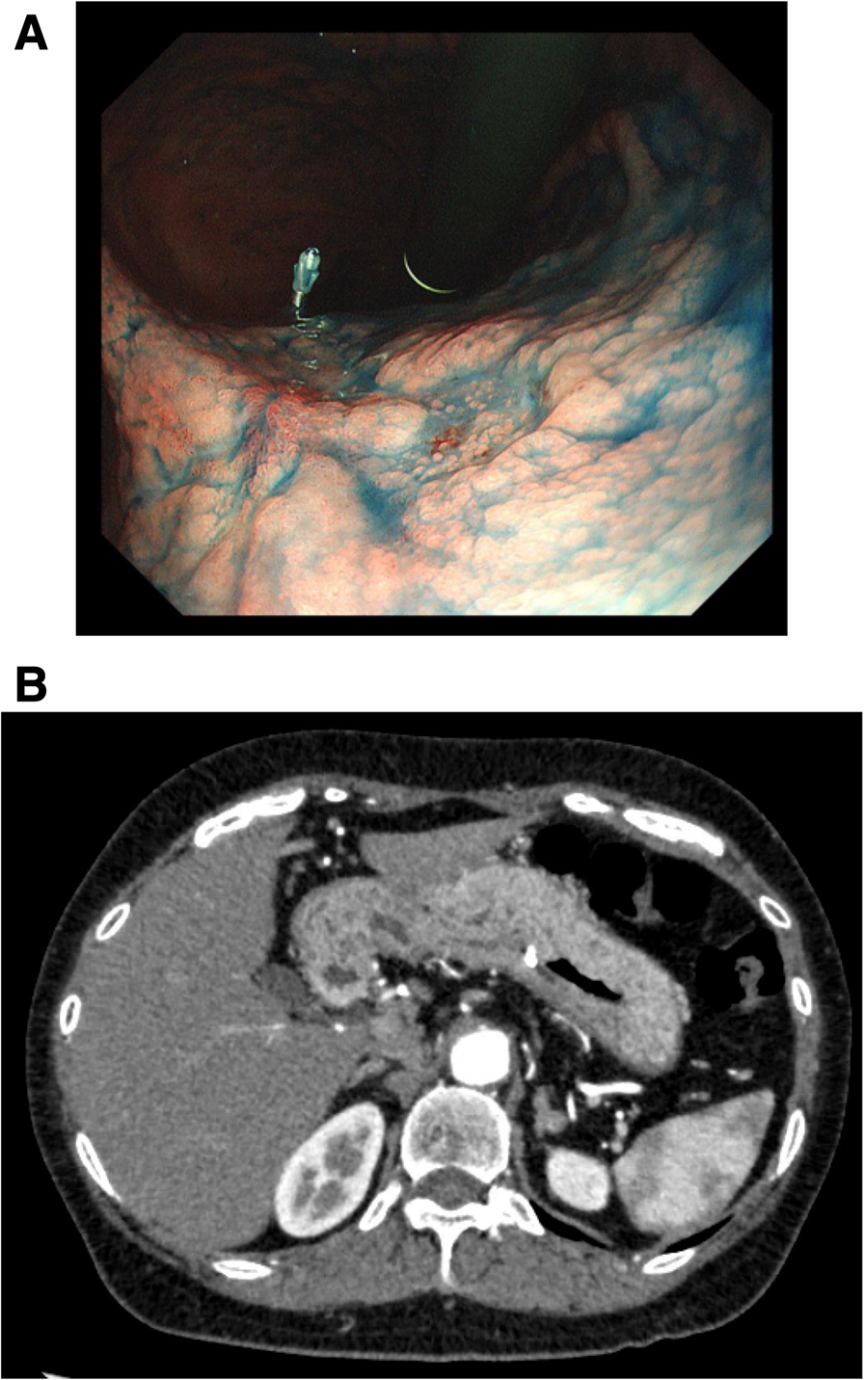
Gastric endoscopy and CT. **a** Gastric endoscopy revealed a type 0-IIc tumor, which was located in the posterior wall of the mid-body. Histological findings indicated a poorly differentiated adenocarcinoma. Tumor recession without ulceration indicated tumor invasion deeper into the submucosal layer. **b** There was no lymph node swelling based on CT. The preoperative clinical stage was cT1b, cN0, cM0, cStage I

Physical examinations and blood tests revealed nothing of note, including tumor markers. Esophagogastroduodenoscopy (EGDS) revealed that the type 0-IIc gastric cancer was located in the posterior wall of the mid-body of the stomach (Fig. 1a). The histological type was poorly differentiated adenocarcinoma. EGDS and CT (Fig. 1b) suggested that the depth of tumor invasion was the submucosal layer without lymph node swelling. The clinical stage according to the TNM 7th edition was cT1b N0 M0, cStage I.

Based on preoperative examinations, we planned laparoscopic distal gastrectomy with D1+ lymph node dissection and Billroth-I reconstruction.

**Fig. 2:**
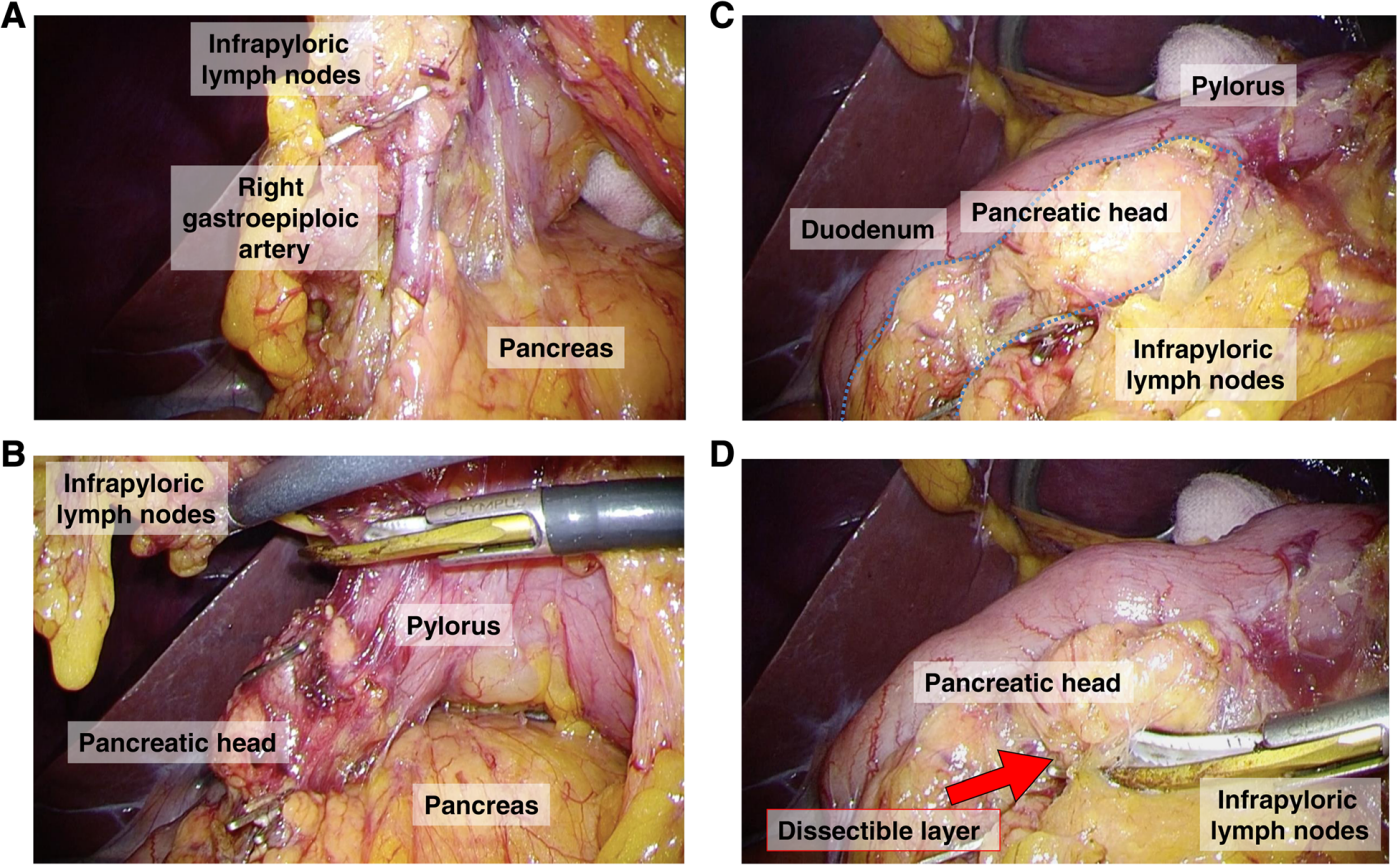
Operative views during infrapyloric lymph node dissection. **a** View of the infrapyloric area. Right gastroepiploic vessels were dissected and cut during the infrapyloric lymph node dissection in close proximity to the pancreas. **b**, **c** A part of the pancreatic head could not be separated from the first part of the duodenal wall during the infrapyloric lymph node dissection. Irregular adhesion of the pancreatic head to the pylorus made it difficult to dissect the infrapyloric lymph node. **d** We performed meticulous lymph node dissection by accurately tracing the dissectable layer, created by adequate countertraction. Subsequently, the right gastroepiploic vessels were cut and infrapyloric lymph node dissection was performed

The surgery progressed without any problems on dissection around the left gastroepiploic vessels. During the dissection of the infrapyloric area (Fig. 2a), a part of the pancreatic head showed unusual adherence to the first part of the duodenal wall (Fig. 2b). This made it difficult to safely separate the pancreatic head from the first part of the duodenal wall (Fig. 2c). Since we should dissect and mobilize the duodenal bulb as long as possible for Billroth-I anastomosis (delta-shaped anastomosis using linear staplers [5]), we converted our reconstruction plan of the Billroth-I method to Roux-en-Y. For safe and accurate lymphadenectomy without causing pancreatic injury, we deliberately focused on tracing the dissectible layer between the pancreatic parenchyma and fatty tissues, including lymph nodes (Fig. 2d).

**Fig. 3:**
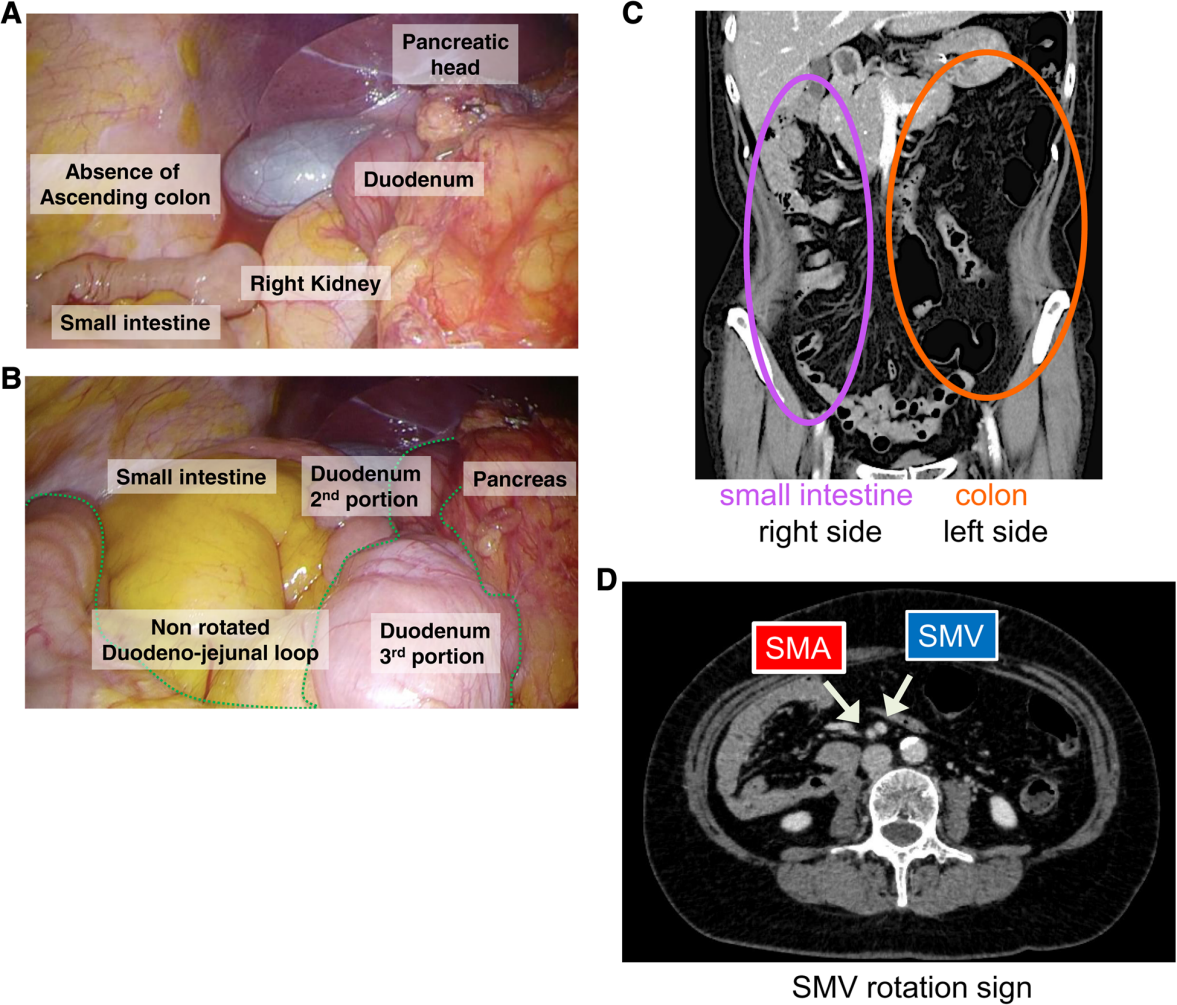
Operative views before reconstruction and CT findings, indicating asymptomatic intestinal malrotation. **a**,**b** There was no right-side colon at the hepatic flexure, and the duodenum did not rotate to the left side of the body. The small intestine was located on the right side of the abdomen, with a completely mobilized duodenum. **c** A right-sided small bowel and left-sided colon sign were confirmed on reviewing the CT image. **d** SMV rotation sign: SMV existed abnormally on the left side of the superior mesenteric artery (SMA)

After distal gastrectomy, we could not find the ligament of Treitz or jejunum on the left side, below the transverse colon. Furthermore, the right-side colon had been completely mobilized to the left-side abdomen (Fig. 3a), and the small intestine was located in the right-side abdomen with a completely mobilized duodenum without duodeno-jejunum loop (Fig. 3b). We reviewed the CT image carefully during surgery, and the right-side small bowel, left-side colon sign (Fig. 3c), and superior mesenteric vein (SMV) inverse position (Fig. 3d) were confirmed. Based on these findings, we diagnosed this patient with intestinal malrotation.

Although we encountered the intestinal malrotation during surgery incidentally, we could complete radical gastrectomy with Roux-en-Y reconstruction laparoscopically. We closed the defect between the Roux limb and retroperitoneum to prevent possible internal herniation. Because there was no Ladd’s ligament, we did not add Ladd’s operation. We also did not perform prophylactic appendectomy due to marked adhesion around the vermiform appendix.

Upon reconstruction, it was straightforward and safe to perform gastrojejunostomy by creating a Roux limb. Furthermore, there was no transverse colon or mesentery behind the Roux limb. Therefore, we closed the defect behind the Roux limb by delicately suturing the Roux limb mesentery and retroperitoneum covering the pancreas. We also closed the mesenteric defect of the small intestine as performed in normal cases. We carefully checked and confirmed that there was no other peritoneal defect.

The type of malrotation was non-rotation (90°). This patient was discharged from our hospital without any complications. According to the TNM 7th edition, the final pathological stage was pT1a, pN0(0/54), M0 pStage IA. Histological analysis revealed signet ring cell carcinoma. After a one-year outpatient follow-up, she was alive without any cancer relapse or complications.

## Discussion

There are 3 divisions of the gastrointestinal tract: the fore-, mid-, and hindgut. During embryonic abdominal development, they rotate counterclockwise 270 ° around superior mesenteric vessels. After this rotation, the duodeno-jejunal loop is fixed on the left side of the midline, which forms the ligament of Treitz.

Intestinal malrotation, which arises from incomplete rotation of the embryonic midgut, is one of the congenital anomalies usually diagnosed in infancy. Wang categorized intestinal malrotation into four groups: non-rotation with incomplete 90 ° rotation, malrotation with 180 ° rotation, reversed rotation, and paraduodenal hernia [4].

Intestinal malrotation occurs in 1 in 500 live births. The most common symptoms are volvulus, intestinal obstruction, internal hernia, and superior mesenteric artery syndrome [4]. When gastrointestinal surgeons encounter symptomatic intestinal malrotation, Ladd’s operation is usually planned, which includes counterclockwise untwisting of the volvulus, division of the abnormal colo-duodenal Ladd’s bands, widening of the mesenteric base to prevent further volvulus, and prophylactic appendectomy [6, 7]. However, most cases of intestinal malrotation are asymptomatic. Patients remain asymptomatic until adulthood without growth disorder. They are incidentally diagnosed with intestinal malrotation on health screening or at the time of treating other diseases such as appendicitis, acute cholecystitis, or abdominal cancer [8–10].

Traditionally, open laparotomy was the mainstay of gastric cancer surgery. However, over the last 20 years, minimally invasive surgery for gastric cancer, such as laparoscopic and robotic gastrectomy, has rapidly developed [2, 11–15]. One of the advantages of laparoscopic surgery, including robotic surgery, is the local magnification effect. On the other hand, as a weak point, it is difficult for the operator to visualize the entire abdominal cavity during laparoscopic surgery. That is one of the reasons why we did not recognize the anatomical anomalies until we began gastrointestinal reconstruction in this case. When we incidentally encounter anatomical anomalies during minimally invasive surgery, such as intestinal malrotation, it is important to review CT images carefully even during surgery to avoid any misunderstanding of surgical anatomy and injury of organs and vessels.

This case showed non-rotation (90 °) without Ladd’s ligament. We performed Roux-en-Y reconstruction with closing the defect between the Roux limb and retroperitoneum to prevent internal herniation, and omitted performing Ladd’s operation. Because adhesion around the vermiform appendix was marked and difficult to dissect, we did not perform prophylactic appendectomy to avoid possible complications caused by unnecessary adhesiolysis.

**Fig. 4:**
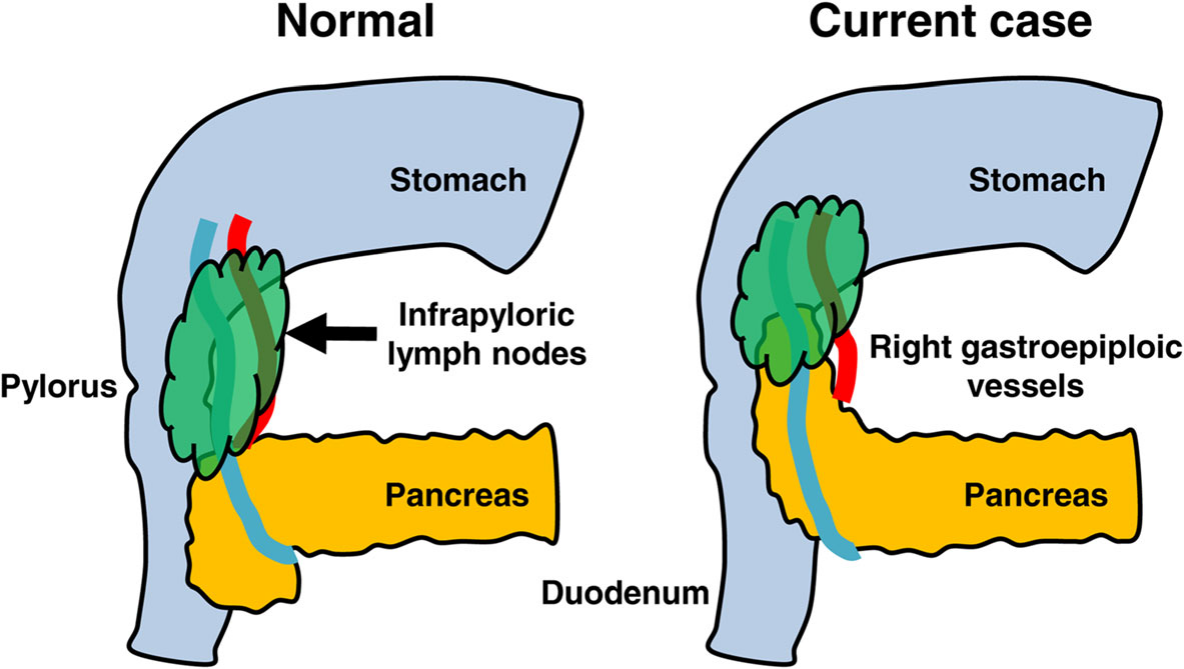
Schema of the infrapyloric lymph node area. The left-side schema represents normal anatomy, while the right-side schema represents intestinal malrotation

There are several reports of gastric cancer with intestinal malrotation described in the English literature [16–24]. Most of them focused on anatomical anomalies and reconstruction. However, it is also necessary to consider oncological matters, as well as the surgical anatomy. During radical gastrectomy with lymph node dissection, one of the most difficult sites is the infrapyloric area. As reported previously, the uncinate process was frequently aplastic or hypoplastic in patients with intestinal malrotation, especially in non-rotation type [25, 26]. This mechanism may have been associated with incomplete rotation of the ventral bud of the pancreatic primordium, which might cause globular and/or elongated pancreatic head. This pancreatic contour abnormality was considered to be associated with abnormal adhesion of pancreatic head to the first part of the duodenal wall. In this case, a part of the pancreatic head showed unusual adherence to the wall of the duodenal bulb, and the distribution of fatty tissues containing lymphatic tissues differed from normal surgical anatomy (Fig. 4). Therefore, there was a potential risk of both pancreatic injury and oncologically inadequate lymphadenectomy. By carefully grasping the lymph node tissue with appropriate countertraction, laparoscopic surgeons should be able to identify dissectible layers for lymph node dissection between fatty tissues and pancreatic parenchyma, even in patients with anatomical anomalies in the infrapyloric area.

## Conclusion

We encountered a patient showing asymptomatic malrotation with gastric cancer who underwent successful laparoscopic distal gastrectomy with Roux-en-Y reconstruction. From the viewpoint of oncology, an anatomical anomaly in the infrapyloric area makes it difficult to perform radical lymph node dissection with satisfactory safety. However, reviewing CT images carefully to reconsider the anatomical anomalies and tracing the dissectible layer accurately with adequate countertraction can facilitate safe and successful surgery.

## Abbreviations

CT: Computed Tomography
EGDS: Esophagogastroduodenoscopy
EMR: Endoscopic mucosal resection
ESD: Endoscopic submucosal dissection
SMA: Superior mesenteric artery
SMV: Superior mesenteric vein
